# Appreciative inquiry in a Norwegian nursing home: a unifying and maturing process to forward new knowledge and new practice

**DOI:** 10.1080/17482631.2018.1559437

**Published:** 2019-01-09

**Authors:** Inger-Lise Magnussen, Johanne Alteren, Terese Bondas

**Affiliations:** a Faculty of Nursing and Health Sciences, Nord University, Bodø, Norway; b Faculty of Nursing and Health Sciences, Nord University, Mo I Rana, Norway

**Keywords:** Appreciative action research, sensory garden, nursing, participation, knowledge development and culture

## Abstract

**Purpose**: Appreciative inquiry (AI) studies have proven to be useful in developing nursing knowledge and changing nursing practice. However, few AI studies have examined the meaning of participation over time among collaborating healthcare providers. Our aim was to explore and illuminate healthcare providers’ participation over time in a Norwegian nursing home to develop new knowledge and practice, focusing on sensory gardens.

**Method**: Twenty healthcare providers participated in the 3 year AI study. Data were collected in fieldwork, interviews, and interventions. Saldañas’ longitudinal analysis was applied.

**Results**: The collaboration between the researcher and participants created insight of a relational room, which was named “the room of closeness”. Participants’ search for new arenas to apply the meaning of the room of closeness was found when focusing on the sensory garden. Their desire for joint development created a bottom–up perspective, the hallmark of successful AI.

**Conclusion**: Knowledge of participants’ experiences may contribute to developing AI as a useful and transferable method, especially regarding co-creating participation, and may have implications for research and society. AI’s strength-based approach may, however, lead to the neglect of data that are associated with problems, and complicate the assessment of success. Further research is therefore needed to develop AI.

## Introduction

Health and nursing research has shown increased interest in participatory research methods. Value is appraised by new knowledge; however, the changes have been based on participants’ voices and equality in power structures related to education and position (Balbale, Locatelli, & LaVela, 2016). Appreciative inquiry (AI) has the potential to mobilize healthcare providers’ intentions and joint involvement in developing knowledge of sensory gardens in a nursing home setting (Magnussen, Bondas, & Alteren, 2016), and to present a unique opportunity for finding new insights in collaboration with healthcare providers, such as creating “the room of closeness” (Magnussen, Bondas, & Alteren, 2017).

A “sensory garden”, as in this study, was defined as a carefully planned, fenced, and cultivated outdoor space used in caring for patients with dementia, in order to reduce symptoms, provide patients with the opportunity to cope, and facilitate contact with nature in a safe environment (Berentsen, Eek, & Grefsrød, 2007). “Appreciative” was defined as “being conscious” of both one’s own and others’ experiences, ideas, and reflections (Cooperrider & Srivastva, 1987). The inclusive nature of AI contributes to collaboration and facilitates workforce engagement that promotes changes in a healthcare context from a bottom–up perspective (Trajkovski, Schmied, Vickers, & Jackson, 2013b). AI provides a positive and new way of participating in healthcare and health research, which is often described as engagement, involvement, and inclusion (Trajkovski, Schmied, Vickers, & Jackson, 2013a), and is tied to open and trusting relationships that contribute to continuous changes (Bondas, 2009).

In this study, AI was perceived as a strength-based approach where feeling hope, being future oriented, and having visionary thinking were the basis of new successes related to better care (Cooperrider & Srivastva, 1987). Inspired by social constructivism, AI is based on the idea that reality can be described and understood in various ways, and that the valuation of one’s own practice can increase its value (Whitney & Trosten-Bloom, 2010).

### Previous studies on AI in older people’s healthcare

AI is considered a useful and powerful method to improve healthcare providers’ communication skills and professionalism in nursing homes, through promoting supervision, training, and reflection (Wadensten, Engholm, Fahlström, & Hägglund, 2009). Hospital employees have experienced AI’s contribution to increased involvement in decision-making processes, and enhanced sensitivity to cultural differences (Havens, Wood, & Leeman, 2006), as well as improved care for patients with dementia, when focusing on good encounters between the healthcare provider and the patient (Scerri, Innes, & Scerri, 2015). By also involving patients and relatives in the AI process in hospitals, practices that had been taken for granted were challenged, replacing uncertainty with openness and confidentiality (Dewar & Nolan, 2013), and facilitating the implementation of new practices (Dewar & Kennedy, 2016). Participant involvement can be missed if AI criteria, such as a democratic process, equality, and voluntary participation, are not employed in research (Watkins, Dewar, & Kennedy, 2016). Several studies consider leaders as key figures in changing processes, and they emphasize the importance of management support in enabling the application of AI (Dewar & Nolan, 2013; Shield, Looze, Tyler, Lepore, & Miller, 2014).

Action research (AR) is an overarching umbrella within participation research, which enables multiple approaches (Williamson, Bellman, & Webster, 2012). Traditional AR, which focuses on problems, compared to AI’s focus on promoting continuous improvement, is useful in nursing research, including the personal development of participating nurses (Bergdahl, Benzein, Ternestedt, & Andershed, 2011). AR promoted a gradual change in attitudes towards patients and in practice in nursing homes in Norway; however, a complete cultural change was hampered because the entire staff did not participate in the guidance, lectures, and reflection (Lykkeslet, Gjengedal, Skrondal, & Storjord, 2014). A Danish AR study concluded that it was uncertain what each individual participant had achieved, even though participants were engaged when they had real influence in decision making (Teglborg, Hovdenak Jakobsen, & Kragelund, 2015).

Involving and empowering diverse groups of participants is common in AR, as can be seen in a participatory action research (PAR) study, which focused on food distribution to elderly residents living at home in Sweden (Pajalic, Persson, Skovdahl, & Westergren, 2012), and in participatory and appreciative action and reflection, which focused on meaningful daily life for older people in nursing homes, also in Sweden (James, Blomberg, Liljekvist, & Kihlgren, 2015; James, Fredriksson, Wahlström, Kihlgren, & Blomberg, 2014). A follow-up study showed that participants’ fear of conflicts, lack of openness and trust, and the time period, may lead to successful cooperation becoming limited (Juthberg & Ericson-Lidman, 2016). PAR appeared feasible for collaboration between staff and patients in nursing homes in Belgium by combating traditional thinking, and participants were made aware that changes would be a process of maturation, which required time (Van Malderen, De Vriendt, Mets, & Gorus, 2016).

Participation in AI has shown that new knowledge occurs through interaction with participants, as the researcher cannot create an AI alone (Magnussen et al., 2016, 2017). AR’s results in relation to process and participation often appear to be linked to concrete actions and described as “before and after” (Nyman, Bondas, Downe, & Berg, 2013), while the “path” that participants have taken appears to be less studied. Participation is fundamental for the development of knowledge in AI, and there is a need to explore and highlight the participation itself over time to develop such knowledge. Several AI studies have shown that participants often become creative and engaged in the beginning; however, little is known about how the process evolves (Reed, 2010). When applying an AI research design, we know less about how this participation is co-created and what the meaning is of the participation over time. The current study may contribute novel knowledge, especially in a nursing home context, which could have relevance for vulnerable patient groups in other nursing contexts. In this study, participation was the healthcare provider’s engagement and involvement in the AI process, and was part of a research programme developing new knowledge and promoting the development of the sensory garden in nursing homes.

### Aim and research questions

The aim was to explore and illuminate healthcare providers’ participation over time when developing new knowledge and new practices in an AI study. The context was a Norwegian nursing home, focusing on the development of a sensory garden. We posited two research questions: (1) What is healthcare providers’ participation over time in an AI research process? (2) How has participation in an AI process in a nursing home contributed to developing new knowledge and new practice from a healthcare provider perspective?

## Design and method

### Appreciative inquiry

An AI was chosen for this study. The nature of AI calls for collaboration, contributes to the creation of good relations and results (Dewar & Nolan, 2013), and also ensures the development and evaluation of practices (Whitney & Trosten-Bloom, 2010). The AI process promotes development based on the healthcare provider’s practical knowledge and vision of what they want to change, and a participatory approach provides the opportunity for changes from a bottom–up perspective (James et al., 2015). AI’s hallmark and value in uniqueness, wholeness, and humanity coincides with the characteristics of nursing, such as care, mercy, and compassion (Bondas, 2003; Dewar, Adamson, Smith, Surfleet, & King, 2013), and supports the chosen research method in a nursing context. This study is based on Cooperrider’s cyclic 4-D phases: discover, dream, design, and destiny (Havens et al., 2006). The 4-D phases are not rigid steps and can be adapted to the setting and participants (Trajkovski et al., 2013a). The current study used interviews, participatory observation, reflection, evaluation, and process data from the four phases of the AI process in a study of a sensory garden in a Norwegian nursing home.

### Context and participants

The study was conducted at a nursing home in a municipality in northern Norway. The context was a nursing home ward with its own sensory garden, which is named the sensory garden ward (SGW). The SGW accommodates six residents with moderate to severe dementia. It is one of three wards in the nursing home, which has 38 full-time employees and 30 patients. The SGW is staffed by two employees on day and afternoon shifts, respectively, and shares night-shift personnel with the nursing home. All employees at the SGW were invited to take part. Of the 20 participants—all women, with a mean age of 47 years—five were nurses (one of them also had management responsibilities), eight were assistant nurses with other educational backgrounds, and seven were healthcare providers without formal education.

In Norway, nurses obtain a 3 year university education, and nursing assistants obtain a 1–3 year education from a high school or trade school. Norwegian nursing homes are usually staffed with a variety of nursing staff; therefore, the selection of participants in the study was representative. All staff work closely with the patients and participate in their daily care; the nurses also have a professional responsibility. Owing to sickness and leaves of absence, there were some changes in the group of staff members participating. By including new staff, there were always 12 healthcare providers and two leaders involved in the project. Ten individuals participated the entire time. Caring staff at the SGW were termed “healthcare providers”.

### Ethical considerations

The research plan was sent to and advised upon by the Norwegian Centre for Research Data (1 January 2014), the Norwegian Regional Committees for Medical and Health Research Ethics (1 November 2014), and the municipality (5 August 2014). Informed consent was obtained, and a description of the research, anonymity, confidentiality, and the possibility of withdrawal were all given, along with the opportunity to read and rectify all collected information before publication. Trust, respect, and candour were emphasized, all of which can, as stated by Parkin (2009), prevent uncertainty, frustration, and vulnerability among participants, and can maintain anonymity and confidentiality in an AI fellowship. The SGW, which houses people with dementia, was the research site and the nursing home manager provided information about the researcher’s presence in the SGW to both patients and their families.

### The AI process

The participants’ engagement and involvement throughout the AI process and the researcher’s guidance and support promoted the implementation of AI in context. In the early phases of the project, participants were introduced to an appreciative way of thinking (Magnussen et al., 2016). The participation and future planning were both positive and confirming, which is the essence of AI, according to Whitney and Trosten-Bloom (2010). In the last phase of AI, the decision to be made was how to implement new knowledge and make lasting changes (James et al., 2015). Challenges with structures, which can be used to establish new practices, characterize this last stage as the destiny phase, according to Cooperrider’s cyclic 4-D phases: discover, dream, design, and destiny (Havens et al., 2006). The destiny phase is about letting the AI work so that relationships with others and with reality can create new patterns, structures, and culture. The healthcare provider’s participation is presupposed.

Data were collected throughout the AI process by both the researcher and participants. The participants’ relationship with nature and the garden, the use of the sensory garden, and their wishes and visions, expressed through individual interviews, formed the baseline of the study (Magnussen et al., 2016). Participatory observation was conducted in the SGW and out in the sensory garden, observing the patient–healthcare provider relationship when focusing on sensory gardens, over a period of 2.5 years, for a total duration of 500 h. During this period, four consecutive interventions were co-created and developed in practice: (1) making practice visible through words; (2) trying out new knowledge of the room of closeness; (3) workshops; and (4) developing knowledge of the room of closeness. Three workshops were planned and implemented in collaboration with participants, and they followed and supported the development of the interventions, which is described in a previous study (Magnussen et al., 2017). The healthcare providers took part in groupwork and took charge of role-playing, which demonstrated previous, present, and desired situations in daily care. The evaluation survey, using a Likert scale ranging from 1 to 6, where 6 represented agreement with the assertion and 1 not in agreement, was conducted in connection with each workshop gathering. Appreciative reflection, based on the healthcare providers’ experiences, took place regularly throughout the project period.

### Analyses

To analyse the data relating to participation over time, a longitudinal qualitative analysis method was chosen (Saldaña, 2003). Rich and varied data, which consisted of text and completed surveys, were divided into periodical data sets to identify the changes and how they had occurred and developed. First, the data were read and reread, with the research questions in mind. In the next stage of the analysis process, meaningful units were identified from the starting interview that were connected to the healthcare provider’s participation. These units generated themes and questions, which were illustrated by data from the distinct data sets. A change map was introduced as an aid in this process, and themes and subthemes were developed. The sets of data were not seen as separate and loose, but were intertwined throughout the entire analysis process, and the analysis of the data and changes through time will thus appear fluid (Saldaña, 2003). The analysis was performed by the first author in close collaboration with participants, and was discussed with the coauthors, who acted as a reference group.

## Results

The following three themes were found through the analysis process: (1) co-creating a new path—not just the beginning and ending; (2) shared experiences bring back both co-creation of new knowledge and the room of closeness; and (3) the room of closeness—a new domain in nursing.

At the beginning of the AI project, the healthcare providers communicated will, engagement, and positivity in taking part in developing the sensory garden. The SGW had no master plan for its development and use, and several of the healthcare providers saw this as both a challenge and a restriction for developing the sensory garden. In the first phase of the project, a new way of being in the SGW together with the patient was discovered: creating the room of closeness. “The room of closeness” is defined as a sensory garden–patient–healthcare provider relationship, where the sensory garden as a medium helps to create calm, close attention, security, equality, and recognition. The sensory garden became an abstract place to meet and get to know the patient as a person. The healthcare providers considered the room of closeness as a significant relationship in care. Knowledge of the room of closeness was converted into caring action, and was gradually implemented in the routines of the SGW. Through the knowledge of the room of closeness, the healthcare providers saw their own behaviours, the interaction between them, and the care of the patients in a new way. To ensure anonymity in the presentation of the results, pseudonyms were used, and Groups 1, 2, and 3 represent the workshop groups.

### Co-creating a new path—not just the beginning and ending

The healthcare providers’ wish to develop the sensory garden as a joint project would give all participants the possibility of taking new initiatives and co-creating knowledge. Recognition of the healthcare providers’ practice, knowledge, and vision creates an opportunity for involvement, and both motivating and challenging accounts along the path are described. AI is not a straight road, but a path that is created as the AI unfolds. This theme is illustrated by two subthemes: (1) will, courage, and honesty—door openers for collaboration and joint development; and (2) getting to know each other in relationships—a maturing process.

#### Will, courage, and honesty—door openers for collaboration and joint development

In the initial interviews, healthcare providers communicated interest and curiosity regarding the project. Tove said, “I am positive, excited, and open, and I am happy to gain new knowledge”. Several healthcare providers wished for collaboration towards the common goal of increasing the use of the sensory garden and providing the very best care for the patients. Åse said, “We have visions, and we do not want to stop now”, and some healthcare providers said that “we will cross the bridge when we reach it”. Many of the healthcare providers had expectations of the project; Sissel said, “I wish to know about the project’s progress and development—not just a start and a stop. It’s exciting to follow how things progress”.

Lack of time hindered several healthcare providers’ engagement throughout the period of the project; Beate felt frustrated and lost interest. Turid felt that she had not been very involved and felt no continuity, while Åse felt that she was informed and involved in the project work. Tove thought that they had a limited time to work together and to come up with innovative ideas, so the project could provide them with initiative and enthusiasm. Some healthcare providers said that they were uncertain of what they could contribute; as Mari said, “I haven’t worked here for very long and I have no education”. Furthermore, Mette said, after some time, “When the interview was over, I was so sure that this wasn’t for me. I didn’t have the courage to say ‘no’, so I joined, and I have not regretted it”.

During the project, many healthcare providers admitted that they now dare to take part in reflection, more so than before; Åse said, “Healthcare providers, whom I found to be evasive, now participate in the reflection and talk a lot”. The healthcare providers, who in the beginning felt uncomfortable in reflection, gradually allowed the researcher’s proximity, which they ascribed to the researcher’s discreet, calm, and withdrawn manner. Several healthcare providers described difficulties in focusing on the sensory garden over a longer period because of time and work pressure, and many of them said, as Bente did, “The researcher’s presence and motivation helped us keep focus”. Workshops were planned together with the healthcare providers and the management, and Rita, one of the healthcare providers, said, “I wouldn’t mind taking part in role play, and I think I know which part I would want to play”. Management facilitation and involvement in the project motivated and fortified the healthcare providers to make a joint effort. As Kari said, “It’s great to know that they are prioritizing the sensory garden and us”. We can conclude that will, courage, and honesty in the AI open the door for collaboration and joint development.

#### Getting to know each other in relationships—a maturing process

Some healthcare providers found participating in AI foreign and unsafe, and their immediate thoughts and feelings, particularly concerning observation and reflection, are described with words such as uncertain, difficult, unpleasant, foggy, dubious, and scary. Several healthcare providers expressed, as Turid did, “[in the beginning] We are unaccustomed to think about how we use ourselves. Positive feedback is good; it feels good”. Observation showed that healthcare providers who were uncertain tended to back out, especially from participating in reflection. Other healthcare providers reflected together with the researcher, and invited and motivated the uncertain healthcare providers to participate.

The healthcare providers shared and discussed discoveries and ideas in the workshop gatherings. In the last workshop, Group 1 said, “Increased professional knowledge increases confidence in the work, and an increased mutual, professional base makes reflection easier and meaningful. We have become better at seeing what is good”. The healthcare providers experienced both personal and professional development in interacting with each other, which they ascribed to both collective appreciative reflection, and the researcher’s presence and acknowledgement. During the last workshop, Group 3 said, “We feel that the researcher has not been here very much during the past year. We want more repetition of what we have learned and further motivation. It is easy to fall back into our old ways”.

Through reflection and workshop sessions, healthcare providers felt that they were becoming closer; as Kari stated, “I have gotten to know my colleagues much better, and that has made me more aware of the importance of making my co-worker better”. In the second workshop session, Group 1 said, “We now trust our own judgement and actions and advise others not to be afraid of trying new things”. Beate, who seldom dared to take the initiative to read aloud and sing, said, “I took a song book, sat down, and made eye contact with the patient. We talked and sang, held each other’s hands, and rocked to the song. The patient smiled, and his eyes were shining. I dared, and I won”. Beate’s courage is one example of the development of self-confidence, and Mette felt that the reflection had been “a journey” from uncertainty and insecurity to affirmation and confidence.

In the last workshop session, Group 2 said, “We now often emphasize each other’s strengths and give each other credit”. Professional and appreciative guidance from both colleagues and the researcher is described as affirmative and motivating. As Tove said, “The researcher has made me more confident in my job”; Linda added, “The room of closeness is unifying; it’s ours”. Therefore, getting to know each other in relationships is a maturing process in the AI.

### Shared experiences bring back both co-creation of new knowledge and the room of closeness

The healthcare providers’ practices and reflections contributed to the co-creation of the room of closeness, a sensory garden–patient–healthcare provider relationship. The room of closeness engages the healthcare providers and makes it easier for them to express, reflect on, and document what they do. Knowledge of the room of closeness gives the healthcare providers a new understanding of the meaning of the sensory garden and of cooperation in nursing. The person of closeness (the specific healthcare provider creating the room of closeness) and the facilitator (the healthcare provider preserving the room of closeness) are valued as equal parties. The theme is illustrated in two subthemes: (1) the room of closeness—a room for awareness, sensitivity, and new insight; and (2) the room of closeness—a room for teamwork, equality, and joy in caring

#### The room of closeness—a room for awareness, sensitivity, and new insight

In the beginning, four healthcare providers shared their experiences and reflected on what happened and why, and what they did and did not do when discovering and naming the room of closeness. As Mette explained, “It’s exciting to reflect on the room of closeness. I am more aware of preserving it”. Several healthcare providers discovered that conversations and reflections increased their awareness; as Åse stated, “I have become more aware of how important it can be, for both the patient and the care provider, when creating this intimate and confidential encounter, regarding respect for the patient’s lived life”. The four healthcare providers motivated their colleagues to participate in creating the room of closeness, and described it being easier to reflect on joint experiences. In the beginning, many found it difficult to reflect; as Guri said, “There are other kinds of questions asked, which we are not familiar with. They make me think in a new way, and I like such questions”. In addition, several stated that they adapted to talking about what they do, while other healthcare providers, such as Beate, experienced the following throughout the project period: “I am struggling with expressing things, and I worry about making mistakes or being misunderstood”.

Observation showed that healthcare providers described and discussed factors that they felt either enhanced or hampered the creation of the room of closeness. Guri said that she now notices this room of closeness, as she has more understanding of what it means for the tranquillity and the atmosphere of the SGW. During the second workshop, Group 2 said, “It is important to share experiences of the room of closeness and to learn from each other”. Many healthcare providers found actions and descriptions from others instructive; as Tove said, “Documenting conversations and activities is enlightening. It makes me more focused and responsible”. Furthermore, some of them described how knowledge of the room of closeness gave them a new understanding of nursing, which they felt the researcher’s professional and instructional feedback added to. During the last workshop, Group 2 said, “We have become more aware of how and why things are done, along with an increased understanding of the profession, the dementia illness and the patient’s needs”. We can see from this that the room of closeness creates awareness, sensitivity, and new insight.

#### The room of closeness—a room for teamwork, equality, and joy in caring

Turid said that the room of closeness is a collaboration and requires good interaction between healthcare providers. The healthcare providers felt they were working more as a team, and the dialogue between Trine and Åse is one example of this:

Trine: “I saw the patient searching for closeness and security, and when you connected with Peter [the patient], I chose to stay in the background and went for a walk with the other patients”. Åse: “I got in close contact with Peter. We sang and wandered around, and the closeness, with eye contact and holding hands, lasted throughout the shift, undisturbed. It became good nursing”.

Åse said that she looks differently at the knowledge of what she does when, through reflection and words, she gains insight into what she is doing, and she thinks that this is amusing.

During the project, the healthcare providers discovered that both the person of closeness and the facilitator play an equal role in creating the room of closeness. Several described that they consciously facilitated creating the room of closeness; Turid (facilitator) said, “I saw that calmness and good interaction were crucial for creating a room of closeness”. Many said that they tried to see the situations both from their own and from others’ point of view, and they found that cooperation in general had improved; as stated by Linda, “Before, the shift worked well together with like-minded colleagues; but, now I have satisfactory shifts together with all of them, and we are working towards the common goals”. Kari said that she experienced using herself in a new way which was unknown to her, and Guri put it in the following way: “I have learned to see myself in situations and interactions, for better or worse”.

Several healthcare providers found the room of closeness synonymous with giving the patients proper care; Kari added, “Being able to create the room of closeness gives [me] a feeling of succeeding”. They hoped that their colleagues would succeed and, as Mari and Linda said, “Together we are providing good care in an equal partnership”. Some healthcare providers recounted that when they could not create the room of closeness, or the patient did not respond, they felt resigned and discouraged. Others said that they felt despair and anger when the room of closeness was not respected and protected. Interpersonal chemistry between co-workers can also be an inhibiting factor for cooperation, as mentioned by Åse: “When I feel that I’m not being acknowledged by my colleague, I get frustrated and upset, and it can be difficult to talk about”. In the last workshop session, Group 1 summarized it as follows: “Communication has improved, we are more unified, and the ward is calmer”. In sum, the room of closeness creates teamwork, equality, and joy in caring.

### The room of closeness—a new domain in nursing

Knowledge of the room of closeness and the roles that the person of closeness and the facilitator play gives the healthcare providers a new way of thinking and working. The content and use of the sensory garden, and patients’ previous experiences with gardens and nature, came into focus. The healthcare providers’ wish for an all-year garden and its planned use is limited by, among other things, a lack of resources. The theme is illustrated by the following two subthemes: (1) the room of closeness—creating extended use of the sensory garden; and (2) the room of closeness—new arenas for using the sensory garden.

#### The room of closeness—creating extended use of the sensory garden

Tove said, “The room of closeness is becoming a part of the care, it’s here, and it’s here to stay”. The room of closeness was used in daily care, both inside and outdoors, to maintain calm and to prevent unrest, and to offer the patients protection and positive experiences. As Turid said, “I saw that the patient was feeling unsettled and wanted to leave the ward. I picked up a book of birds. It had text, pictures, and sounds. The patient listened intently to both the reading and the bird sounds (chirping), and this moment created a peaceful atmosphere that lasted”.

Many healthcare providers related that conversations in the room of closeness could be about sad, happy, or funny things, which could be expressed through singing, laughter, and tears. As Kari said, “Missing parents, grieving forgotten words, a shoulder to cry on, a hug, or a dearly loved song makes the room of closeness very small and intimate”. During the last workshop, Group 1 explained, “It is important to be attentive to, and preserve, the patient’s resources, and strengthen the patient’s identity”.

Based on observation, the room of closeness appeared to create a homelike atmosphere, where routine was placed in the background. The healthcare providers stated that knowledge of the room of closeness was critical in individual care, and some suggested obtaining information about what the patient likes to do outside when they move in to the ward. During the last workshop, Group 2 summed it up as follows: “It’s important to know what the patients are able to do and what they want to do, and to have information of their former homes to be able to talk about this”.

During the final year, new knowledge of the room of closeness was established in written routines. The planning and organizing of daily activities, including the use of the sensory garden, took place at the start of each shift. Reflection on the execution of activities and creating the room of closeness occurred at the shift’s end. Many healthcare providers found that the routine gave structure to the work day, which made it more predictable. Others found it hard to plan because of situations, such as disturbances in the ward, or different priorities among the healthcare providers. As Tove said, “We didn’t plan well in the morning; so, the day was spent tidying the kitchen and patient rooms. We ought to decide on the activity of the day and make time for reflection”. Guri and Rita helped to motivate each other and said that cooperation is key for the “good encounters” and work is meaningful for both the person of closeness and the facilitator. The room of closeness created extended use of the sensory garden.

#### The room of closeness—new arenas for using the sensory garden

The healthcare providers said that the room of closeness gave them the opportunity to get to know their patients’ interests and connection to nature, gardens, and places. Linda learned this while showing pictures to one patient from the spring flower catalogue: “Several of the patients recognize flowers and bushes, and they talk about plants in the sensory garden and past berry-picking excursions”. When the patients were given the opportunity to smell and taste the chives that Mette had brought in, several of them remembered chives from their own gardens. Beate said, “Mary [patient] told me that she used to put chives in the butter to make it extra tasty when having guests. Some day we can do that”.

At the beginning of the research project, the healthcare providers had visions and ideas for the sensory garden and wished for, among other things, strawberries, raspberries, potatoes, radishes, herbs, chickens, perennials, games, music, a shelter from the wind, raised flowerbeds, and tall trees. Kari stated, “Maybe we can find other arenas for using the sensory garden”. Many of them also wished to have year-round use of the sensory garden and the grill hut, along with access to outdoor clothing, and recognized that this would require money, knowledge, labour, and time, in addition to planning.

Some healthcare providers initiated the realization of these dreams. Specifically, Guri and Beate arranged an autumn gathering in the grill hut, despite the rain. Turid stated, “I bought flower seeds, which I sowed indoors in the spring together with the patients”. The sensory garden is relatively new, and several of the healthcare providers said that they have taken part in planning, establishing the garden, and performing voluntary work. Some described visits to other sensory gardens to get ideas and inspiration, and both Tove and Bente talked about wonderful gardens with berry shrubs and animals.

During the project, the sensory garden’s content was unaltered, with only some shrubs having been moved. Earlier visions for further development of the sensory garden were brought to life during the last workshop session, as shown by Turid saying, “I will check if we can have rabbits in the sensory garden, and I think some of the patients would like to help feed them”. In addition, Group 1 wrote, “We wish for a cosy and inviting grill hut with curtains and a planting of perennials”. In sum, the room of closeness brought with it new arenas for using the sensory garden.

## Discussion

The aim of this study was to explore and illuminate healthcare providers’ participation over time when developing new knowledge and new practices in an AI study. The context was a Norwegian nursing home, and the focus was on the development of a sensory garden. The healthcare providers in this study demonstrated strong beliefs and attitudes towards developing the sensory garden and patients’ care together, and they were geared towards it. Will, courage, honesty, and joy all opened the door, and were a driving force for reflection, input, and interaction, where the healthcare providers influenced and contributed as a part of something bigger. Participation became an interaction between giving and receiving feedback, recognition, and knowledge, and healthcare providers took responsibility for each other’s development and ability to succeed. Participants’ practices and visions acted as compass needles in the AI process, where interventions were created, planned, and implemented. Through their words and descriptions of their own experiences, new knowledge was discovered, and there was a formation of the room of closeness, which provided an additional use of the sensory garden in nursing. The healthcare providers’ joint efforts contributed to developing collaboration and confident social interactions, and the evolvement of new methodological knowledge about AI for developing nursing knowledge.

### Participation in AI—a maturing process

During the project, healthcare providers became more participatory while sharing experiences, ideas, and emotions in an appreciative manner. Although appreciative ways of thinking and talking felt unfamiliar and difficult at the beginning, engagement and creativity emerged in line with Whitney and Trosten-Bloom (2010), whereby the human potential was realized. This may be attributed to mutual confidence and trust, which is supported by Cooperrider and Srivastva (1987), who stated that good relationships are created when AI is working. Furthermore, other studies have suggested that getting to know each other in relationships seems to promote motivation and participation (Dewar & Kennedy, 2016; Teglborg et al., 2015; Vedsegaard, Schrader, Rom, & Scheel, 2016). Through walking the path together, participation seems to ease changing processes and, in line with previous research, affirmation and acknowledgement are important in promoting participants’ engagement in knowledge development and the changing of practices (James et al., 2015, 2014; Magnussen et al., 2016; Trajkovski et al., 2013b).

The discovery and development of new knowledge, such as the room of closeness, resulted in a turning point for healthcare providers, and caring became both visible and important, as described in depth in previous studies of this research programme (Magnussen et al., 2016, 2017). The healthcare providers gradually dared to try out new knowledge, such as when Turid brought the bird book and Beate sang with the patient. The healthcare providers’ participation in AI is therefore connected directly to patients’ daily care. Observation of and closeness to participants make it possible to follow up with individual participants’ achievement in the project, in both a personal and a professional sense, which other studies have suggested as being challenging (Reed, 2010; Wadensten et al., 2009).

Healthcare providers’ individual progress was described as growth and maturation, which was in line with previous research (Bergdahl et al., 2011; Van Malderen et al., 2016). The uniqueness of this study is that the healthcare providers also developed as a holistic group, and the fact that the project occurred over time is critical to its unifying development. Participants developed a moral responsibility towards each other, which emerged clearly during the study. Applying new knowledge and succeeding in nursing care may be linked to AI’s values including collaboration, applicability, and wholeness (Whitney & Trosten-Bloom, 2010). Healthcare providers’ care for each other and for patients when creating the room of closeness was in line with Bondas’ (2003, 2009) theory of caritative leadership, which focuses on caring for both patients and healthcare providers, and on interactive relationships and being open to knowledge.

Since healthcare providers’ voices were valued in their relationships, which fortified their identity and self-worth, their attention and sensitivity to each other increased, in line with previous research (Havens et al., 2006; Juthberg & Ericson-Lidman, 2016). Furthermore, their insight and consciousness, relating to themselves, co-workers, and patients, seemed to contribute to a more attendant collaborative environment in the SGW. This is supported by Cooperrider  & Srivastva (1987), who found that AI had a consciousness-raising effect. The healthcare providers were proud and felt ownership of the room of closeness, which can be seen in relation to AI’s ability to unite rather than divide (Whitney & Trosten-Bloom, 2010). By participating, they showed courage and strength in becoming confident practitioners, supported by the idea of reflective and empowered practitioners (Pajalic et al., 2012). This study showed a developing process over time when it comes to participation in AI, rather than before-and-after descriptions (Nyman et al., 2013).

### Participating in AI: when closeness leads to openness

The findings show that, in participating in AI, healthcare providers achieved positive changes in their daily work, in both content and collaboration. Getting involved in developing and improving practice was positive for all participants; therefore, caring for the patients is now more meaningful and joyful. This development might be linked to AI’s focus on positive changes versus AR’s focus on problem solving (Williamson et al., 2012). The room of closeness and appreciative reflection are now implemented in the SGW routines. Although not all healthcare providers felt that the routine was feasible, they motivated each other in planning and cooperation. The formation of the room of closeness involves cooperation between the person of closeness and the facilitator, and it becomes a new way of organizing practice. In this collaboration, healthcare providers become equal parties, regardless of position and education, and equality and power balance are deemed critical for change (Balbale et al., 2016).

Healthcare providers felt that they were important to each other, and they were willing to change themselves for the sake of others. This “other-oriented” force is a key step towards balance in power structures and positive change in processes, which seems to require a certain level of personal insight (Whitney & Trosten-Bloom, 2010). Healthcare providers saw that they could make a difference in their relationships with others, as when Trine and Åse both sought the room of closeness. However, they also experienced, as Whitney and Trosten-Bloom (2010) described, a true balance of power, and this may be why they gradually dared to become more involved. Balance of power may also be one of the reasons that the healthcare providers felt motivation and joy in coping; according to Cooperrider and Srivastva (1987), people who have equal opportunities to influence can flourish. Healthcare providers’ uncertainty and doubt about participating, and Mette’s lack of courage to report such doubts, may be a sign of a power imbalance, which an external researcher can both cause and influence (Balbale et al., 2016).

The healthcare providers associated the room of closeness with good care, where the patient was seen and appreciated, which is in line with previous research (Dewar et al., 2013; Dewar & Kennedy, 2016; Dewar & Nolan, 2013; Magnussen et al., 2017). The unique aspect of this study is that nature, via the sensory garden, is a vital component for developing new knowledge for improving practice and for changing culture. Organization and goodwill from the municipality and the nursing home management contributed towards the application of AI. Previous studies suggest that management support and facilitation are of major importance in the implementation of AI (Dewar & Nolan, 2013) and in ensuring the healthcare provider’s leeway for participation (Bondas, 2003, 2009). In this study, implementation of AI also appeared to be caused by the individual healthcare providers’ wishes to facilitate their colleagues’ success in caring, as well as their close relationships with each other and with the researcher. The closeness of these relationships paradoxically opens the possibility of more flexibility, where the healthcare providers may use personal and professional knowledge in innovative ways, taking responsibility for each other and for cooperation. Their positivity and interest in participating, and being considered worthy for participation, seem to constitute the power of willingness. In sum, AI can be a powerful tool that supports genuine participation in the improvement of nursing (Wadensten et al., 2009), and may be juxtaposed with other methods facilitating successful collaboration (Dewar & Nolan, 2013; James et al., 2015; Scerri et al., 2015).

Inconsistent use of AI may lead to loss of participant involvement (Watkins et al., 2016) and, according to Lykkeslet et al. (2014), may hamper lasting changes and cultural change. The degree of participatory involvement will influence the degree of the success of the AI process; the higher the number of individuals involved, the better the basis for change of care (Trajkovski et al., 2013b). In summary, this study revealed AI’s dissolving effect as related to roles and power, which seems to have significance for participation in genuine and viable changes (Whitney & Trosten-Bloom, 2010).

### Methodological considerations

AI appears to have contributed to developing new knowledge and the changing of practice, based on healthcare providers’ everyday knowledge, wishes, and capacity for change; this corresponds with AI’s aim to generate new knowledge and changes in close collaboration with practice (Pajalic et al., 2012; Scerri et al., 2015). As such, this study elucidated AI’s innovative and cooperative nature, which both invites and requires real participation (Trajkovski et al., 2013a). Some healthcare providers, who were involved early on in generating novel ideas, formed a core group, and the other providers were motivated along the path both by the researcher and by their colleagues. By starting from the healthcare providers’ everyday work, with the themes and ideas that are meaningful to them, the findings can be said to emanate from a bottom–up perspective (Whitney & Trosten-Bloom, 2010), and may simultaneously cause a direct impact on the research context (Pajalic et al., 2012).

In addition, the researcher’s opportunity to live in and be part of the SGW strengthens the bottom–up perspective. At the same time, having a presence gave the researcher the opportunity to establish trusting relationships, and participate, guide, provide appreciation, and motivate the data collection and development of interventions through the AI phases.

Evaluation also ensured genuine participation from a bottom–up perspective and strengthened the validity and credibility of the findings. The interventions were created in close collaboration with participants and ensured the development of practice, which strengthens the practice and participatory research approach, as well as the trustworthiness of the findings (Cooperrider, Avital, & Godwin, 2013). The findings and the analysis draft were read and discussed with the participants; this kind of member checking in the analysis process may strengthen participation and the trustworthiness of the findings (Birt, Scott, Cavers, Campbell, & Walter, 2016), and discussions with the coauthors could help to validate the results. Reflection on process validity, democratic validity, and outcome validity was emphasized; AI’s validity was tied to its credibility and utilitarian value (Lincoln & Guba, 1985; Whitney & Trosten-Bloom, 2010). Solid validity may give the results greater transferability and utilitarian value to other contexts, such as nurse education and public planning in healthcare.

The triangulation of data collection methods may have provided the data with a deeper meaning (Parkin, 2009) and helped to minimize the limitations of each method (Dewar et al., 2013). The field notes’ verbose and systematic form may have resulted in data that had particular limitations in comparison with audio and photo recording (Vedsegaard et al., 2016). Longitudinal qualitative analysis offers challenges in providing an overview of comprehensive data, and the division into data sets and use of “changing-maps” could ensure that changes and relationships are discovered (Saldaña, 2003).

Furthermore, the use of a mixed group of participants, with diverse competencies, experiences, and skills, may enrich and add diverse care perspectives. Involvement in the project, from both the management and the healthcare providers, may have contributed to a balance of power and, therefore, led to progress during the research processes (Pajalic et al., 2012). Openness and respect concerning power relations over a longer period may have influenced the development and implementation of new knowledge in a positive manner (Whitney & Trosten-Bloom, 2010). The size of the sample and the perspective of the study, as well as the lack of a comparative basis, may pose a weakness for the study. Patient, next-of-kin, and management perspectives could give insight into the complexity of changing routines and culture (Shield et al., 2014).

The researcher’s presence over time contributed to the development of trust in the relationship with the healthcare providers. The few people who were hesitant in the beginning could remain as bystanders and they could themselves decide when they would possibly attend. They were also given the possibility to attend at their own pace and on their own terms. The honesty and openness, as described by the participants, led to safe relationships and voluntary participation (Whitney & Trosten-Bloom, 2010). Participants’ feedback on certain matters, such as cooperation that sometimes had not worked well, showed the importance of the reflection circle in the AI (Magnussen et al., 2016). Changes could thus be planned and were implemented. The researcher’s supervision in a mode of appreciative reflection opened participants up to self-knowledge and self-criticism. The researcher’s presence in the field may be advantageous; however, it may also lead to the researcher’s role being too participatory. The researcher’s pre-understandings and proximity to the field could mean that data were overlooked; however, concurrently, it contributed to discovering closeness, by being close herself. The researcher’s involvement served as a catalyst for development, movement, and processes, which had not happened without the researcher’s presence over time.

Furthermore, the focus group interviews may not have given the same access to closeness and data. The duration of the study may be considered to strengthen participation, collaboration, and implementation of new knowledge, and the development of new knowledge could be a confirmation of participation and a successful AI process. The appreciative approach can be challenging since employees often wish to discuss troubles and problems (Havens et al., 2006). The strength-based and flexible nature of AI, as well as the lack of consistency and firmness, could lead to questions concerning the study’s scientific basis and limit the evaluation of the study’s success (Trajkovski et al., 2013a, 2013b). To the best of our knowledge, to date, there have been no AI studies showing participants’ development over time; therefore, the findings from this study contribute to knowledge regarding AI. The study’s transferability to other countries and nursing contexts will require further research.

## Conclusion and implications

In this study, methodical knowledge of the AI process was developed in a sensory garden context. New knowledge of the room of closeness was implemented in the new routines in the SGW. AI enabled a change of culture, in which care affects both the working environment and care for patients living with dementia. The study could contribute to discovering and highlighting additional factors that may affect a successful AI process. Crucial factors in this study seem to be the healthcare providers’ engagement, responsibility, and endurance, and the researcher’s presence over time. The duration of the project seems to be critical for promoting participation, preserving the bottom–up perspective, and continuous improvements, and could nurture additional factors.

AI promoted an attentive and gentle approach among the healthcare providers, and careful, visionary, and reflective practitioners emerged from the study. The use of AI in this study has led to knowledge of, and interest in, other research contexts in caring for vulnerable patients, and is a promising and fruitful method for developing knowledge and joint change of practice. This study contributes to knowledge of participation in AI over time, from a healthcare provider’s perspective. Although the room of closeness and appreciative reflection are established in new routines, this “new” process is in an early stage of implementation. It will need time to work, and should be followed up in future studies.
